# Primary Splenic Lymphoma: An Uncommon Cause of Persistent Fever Diagnosed by Examining the “Forgotten Organ”

**DOI:** 10.31662/jmaj.2020-0121

**Published:** 2021-04-02

**Authors:** Hirohisa Fujikawa, Ayaka Kokita, Keisuke Watanabe, Minoru Saito

**Affiliations:** 1 Department of Medical Education Studies, International Research Center for Medical Education, Graduate School of Medicine, The University of Tokyo, Tokyo, Japan; 2 Department of Internal Medicine, Suwa Central Hospital, Nagano, Japan; 3 Department of Endocrinology, Diabetes and Geriatric Medicine, Akita University Graduate School of Medicine, Akita, Japan

**Keywords:** primary splenic lymphoma, forgotten organ, spleen, splenomegaly, multiple splenic hypodense foci, contrast-enhanced computed tomography, fever

An 88-year-old woman was hospitalized with a 1-day history of fever. Laboratory test results demonstrated a platelet count of 6.3 × 10^9^/L and a lactate dehydrogenase level of 314 U/L. Abdominal ultrasound showed gallstones. Although initially cholecystitis was suspected and cefmetazole was administered, the fever lasted for 1 week. Contrast-enhanced computed tomography (CECT) revealed enlarged spleen, multiple ill-defined splenic hypodense foci, and no lymphadenopathy (Figure 1). Considering the CECT findings (i.e., splenomegaly and multiple splenic hypodense foci with ill-defined margins), the differential diagnosis included primary splenic lymphoma (PSL) or sarcoidosis ^(1)^. An ultrasound-guided splenic core needle biopsy was performed, which led to the diagnosis of diffuse large B-cell lymphoma (Figure 2). Subsequently, the patient was transferred to another hospital for treatment.

**Figure 1.**
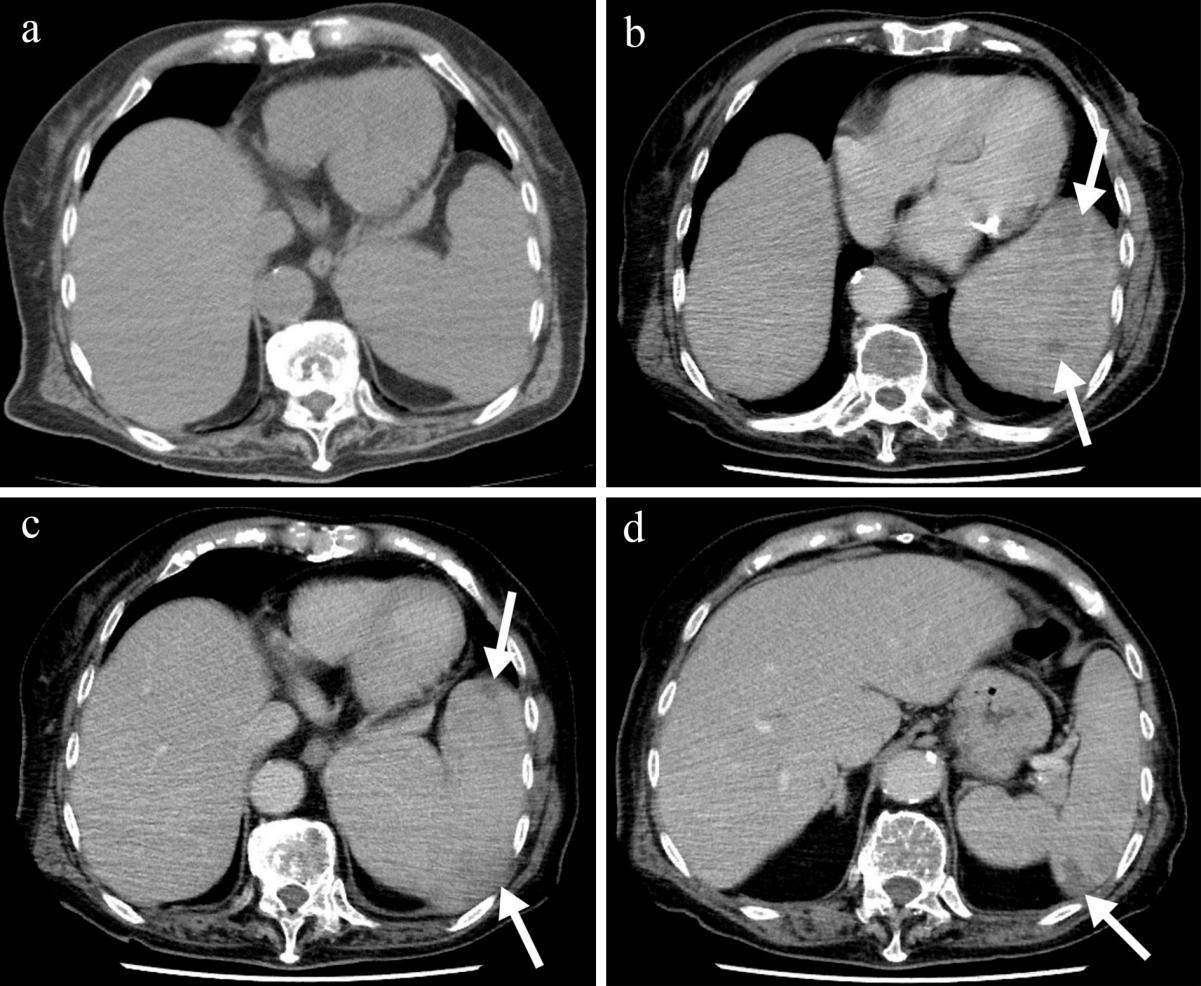
a. Abdominal precontrast computed tomography showing splenomegaly. b-d. Abdominal contrast-enhanced computed tomography showing multiple splenic hypodense lesions and no enlarged lymph nodes.

**Figure 2.**
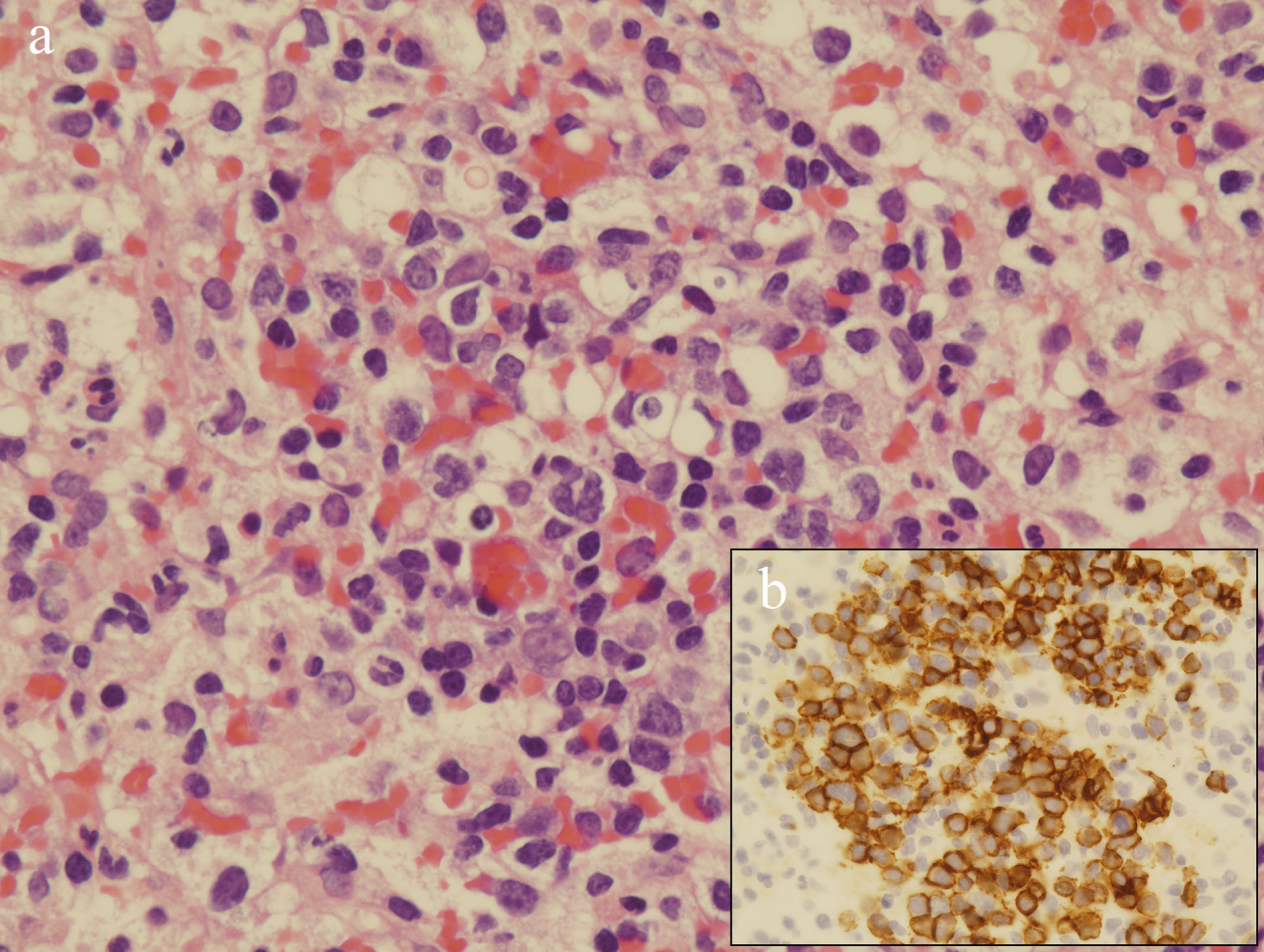
a. Microscopic examination of the splenic biopsy specimen showing diffuse proliferation of atypical cells (hematoxylin-eosin stain; original magnification, 400×). b. Immunohistochemical analysis showing positive CD20 (original magnification, 400×).

The spleen is frequently recognized as the “forgotten organ” among clinicians ^(2)^. However, splenic imaging studies may help establish the diagnosis. In particular, if a patient presents with persistent fever and splenomegaly, both of which are common symptoms in daily practice, clinicians should keep a high index of suspicion of PSL and perform CECT ^(3)^. To arrive at the correct histopathological diagnosis, although splenectomy has generally been performed, splenic biopsy can nowadays be used as an effective diagnostic method with low complication rates ^(4)^.

## Article Information

### Conflicts of Interest

None

### Author Contributions

HF acquired data and drafted the manuscript. AK, KW, and MS reviewed and supervised the manuscript.

### Informed Consent

We have obtained informed consent for this manuscript.

### Approval by Institutional Review Board (IRB)

In this study, IRB approval was not required.
